# Ocular Image and Haemodynamic Features Associated with Different Gradings of Ipsilateral Internal Carotid Artery Stenosis

**DOI:** 10.1155/2017/1842176

**Published:** 2017-06-13

**Authors:** Hui Wang, Hongyang Li, Xiaojie Zhang, Lanyan Qiu, Zhenchang Wang, Yanling Wang

**Affiliations:** ^1^Department of Ophthalmology, Beijing Friendship Hospital, Capital Medical University, Beijing 100050, China; ^2^Department of Radiology, Beijing Friendship Hospital, Capital Medical University, Beijing 100050, China

## Abstract

**Objectives:**

To analyse the changes of ocular haemodynamics and morphology in Chinese patients with internal carotid artery (ICA) stenosis in the current study.

**Methods:**

A retrospective case-control study was conducted with 219 patients. The haemodynamic characteristics, the calibre of retinal vessels, and the subfoveal choroidal thickness (SFChT) were compared. We analysed the correlations with the degree of ipsilateral ICA stenosis.

**Results:**

There were no significant differences among the groups in the central retinal artery equivalent (CRAE), central retinal vein equivalent (CRVE), and AVR (*p* = 0.073, *p* = 0.188, and *p* = 0.738, resp.). The peak systolic velocity (PSV) and end diastolic velocity (EDV) in the central retinal artery (CRA) and the posterior ciliary artery (PCA) were significantly lower than normal eyes (*p* < 0.001). The outer retinal layer thickness and SFChT values of the ICA stenosis groups were significantly lower than normal eyes (*p* = 0.030 and *p* < 0.001, resp.).

**Conclusion:**

The PSV and EDV in CRA and PCA and the SFChT and outer retinal layer thickness of ICA eyes were significantly lower than normal eyes. ICA stenosis may impact choroidal haemodynamics, and decreased choroidal circulation might affect the discordance of the SFChT and the outer retinal layer thickness.

## 1. Introduction

Internal carotid artery (ICA) stenosis/obstruction can lead to severe ischemic cerebrovascular disease. While ICA stenosis may lead to the occurrence of ischemic cerebrovascular disease, ischemic eye disease is another important aetiology that can affect the patient's vision and physical disability and may even lead to blindness and sudden death.

Ischemic ocular manifestations are often the first symptoms in patients with ICA stenosis, 30% of patients with severe stenosis manifested as ocular ischemia syndrome (OIS). ICA stenosis leads not only to nervous system diseases but also to ischemic ocular lesions. Lawrence and Oderich suggest that when the degree of carotid artery stenosis, especially ICA stenosis, is greater than 50%, the incidence of eye symptoms is increased [1]. The reason for this is because intravascular plaque falls off to retinal vascular embolization, vasospasm, and low perfusion. Lyons-Wait found haemodynamic changes in patients with carotid artery stenosis that included retinal vascular obstructions and normal-tension glaucoma, and the incidence of peripheral retinal haemorrhages was increased by 1.8 times, 1.9 times, and 2.4 times, respectively, compared with that of normal people. Furthermore, approximately 5% of patients with severe ICA stenosis will have ocular haemodynamic abnormalities [2]. Chronic progressive ocular ischemia may lead to neovascular glaucoma, optic atrophy, or even permanent blindness [3, 4]. Ocular signs and symptoms secondary to a severe obstructive carotid pathology were first described in 1963 [5] and then gathered under the term ocular ischemic syndrome (OIS) [2, 6]. It is reported that 7.5 out of every 1 million people develop OIS [7]; however, these estimates might be understated, because the links between ocular manifestations and carotid malfunctions often remain unnoticed. So the use of multimodality ocular imaging technology not only is a very necessary way to improve the sensitivity and specificity of diagnosis disease but also plays a role in the early warning of ICA stenosis.

At present, a large number of studies have shown that the retinal vascular calibre is a sign of early systemic microvascular damage signs (e.g., diabetic retinopathy, nephropathy, and cardiovascular disease) [8]. Color Doppler imaging (CDI) is a relatively new technique for quantitatively assessing blood flow velocity [9]. CDI allows a noninvasive examination of blood flow velocities of the ophthalmic artery (OA) and its branches in the orbit, even in states of decreased blood flow such as in OIS [10]. CDI gives important information about the retrobulbar circulation [11]. Arterial hypoperfusion can cause retinal and choroidal microcirculation abnormalities, resulting in insufficient blood perfusion to the retinal inner and outer layers, which may cause ischemic ocular lesions. Therefore, carotid artery stenosis/occlusion can not only affect the retinal blood supply but can also affect the choroidal blood supply situation. At present, the changes in the microstructure of the choroid, especially the choroid thickness, have recently become a new challenge in ophthalmological research. Recent studies have reported that the subfoveal choroidal thickness (SFChT) can be measured noninvasively by enhanced depth imaging OCT (EDI-OCT) [12, 13]. Since choroidal thickness changes are associated with characteristic ophthalmic pathological changes, more studies have been conducted in recent years. Kang et al. reported that patients with OIS have a significantly lower choroidal thickness than that of normal eyes [14].

As early as the 19th century, clinicians discovered there may be a link between carotid artery disease and ocular signs, and carotid artery stenosis can cause ocular ischemic lesions, which has been widely recognized clinically. Our research team has found in the early study that the ocular haemodynamic parameters in patients with carotid artery stenosis had application value in the risk assessment of ischemic cerebrovascular disease [15]. However, for the present research, there was no large sample-related research to confirm this. And there is no correlation research on ICA stenosis that is caused by ocular haemodynamic changes and retinal and choroidal structural changes. Therefore, we have attempted to analyse the relationship between ICA stenosis and ocular haemodynamic changes and to further study the relationships between the ocular haemodynamic changes and choroidal structural changes in 219 people.

## 2. Materials and Methods

### 2.1. Study Subjects and Design

A retrospective case-control study was conducted in all patients with carotid artery malfunctions at Beijing Friendship Hospital, China, from December 2015 to September 2016. We get the information of patients during our data collection. The baseline data was investigated according to the inclusion and exclusion criteria.

#### 2.1.1. Inclusion Criteria

All patients with head and neck CT findings of ICA stenosis or plaque formation were studied.

#### 2.1.2. Exclusion Criteria

Exclusion criteria are the following: (1) presence of refractive error > ±3.0 diopters, choroidal neovascularization or other macular diseases that might affect vision, active intraocular inflammation and/or infection, or a history of any type of intraocular surgery (except cataract surgery) and diabetic retinopathy; (2) patients with peripheral vascular disease, limb paralysis, serious eye disease, and a treatment history (corneal disease, glaucoma, macular degeneration, ocular trauma, pathological myopia, and serious cataract); (3) acute myocardial infarction, stroke within a few weeks prior to this study, and infectious inflammatory disease; (4) persistent abnormal heart rate and chronic cardiac insufficiency; (5) pulmonary hypertension and patients suffering from acute or chronic infectious diseases or malignant tumours; and (6) patients who were uncooperative for evaluations or test results and who underwent carotid endarterectomy before SD-OCT examination could not be analysed.

Patients were selected before baseline data collection and completed the pathology registration form, the contents of which included the following: their general information (age, occupation, marital status, education degree, and working strength); health status (high blood pressure, diabetes, coronary artery atherosclerosis, sex, heart disease, stroke, kidney dysfunction, malignant tumour, peripheral vascular disease, etc.); daily habits (drinking, sleeping, smoking, drinking tea, and physical exercising); eating habits (halophilic, intake of fruits, vegetables, and meat); and family history (first-degree and second-degree relatives suffering from hypertension, diabetes, and coronary heart disease).

### 2.2. Methods

Eyes were divided into four groups with respect to the degree of stenosis. Intravascular ultrasound (IVUS) examination was performed to assess the major ocular vessels (i.e., ophthalmic artery, central retinal artery, and posterior ciliary artery), including the degree of stenosis and haemodynamic changes of the arteries. At the same time, fundus imaging and optical coherence tomography scans were performed to measure retinal vascular diameters, the thickness of RNFL, inner retinal layers, the photoreceptor layers, and the subfoveal choroidal thickness (SFChT). Finally, the correlation between ICA stenosis and retinal blood flow and the correlation between parameters of posterior ciliary arteries (PCA) and the mean SFChT were analysed.

Imaging examinations mainly included the head-and-neck computed tomographic angiography (CTA) inspections and ultrasonic Doppler imaging examinations.

#### 2.2.1. Head-and-Neck Computed Tomographic Angiography (CTA)

The examinations were performed by the Philips iCT (PHILIPS, Netherland). The stenosis ratio was calculated by the NASCET (North American Symptomatic Carotid Endarterectomy Trial) stenosis grading method and was used for the estimated percentage of stenosis and classification. Vascular stenosis degree % = normal vessel diameter (*D*) − narrow vessel diameter (*N*), divided by the normal vessel diameter (*D*) × 100%. The stenosis degree = (1 − *N*/*D*) × 100%.

**Figure 1:**
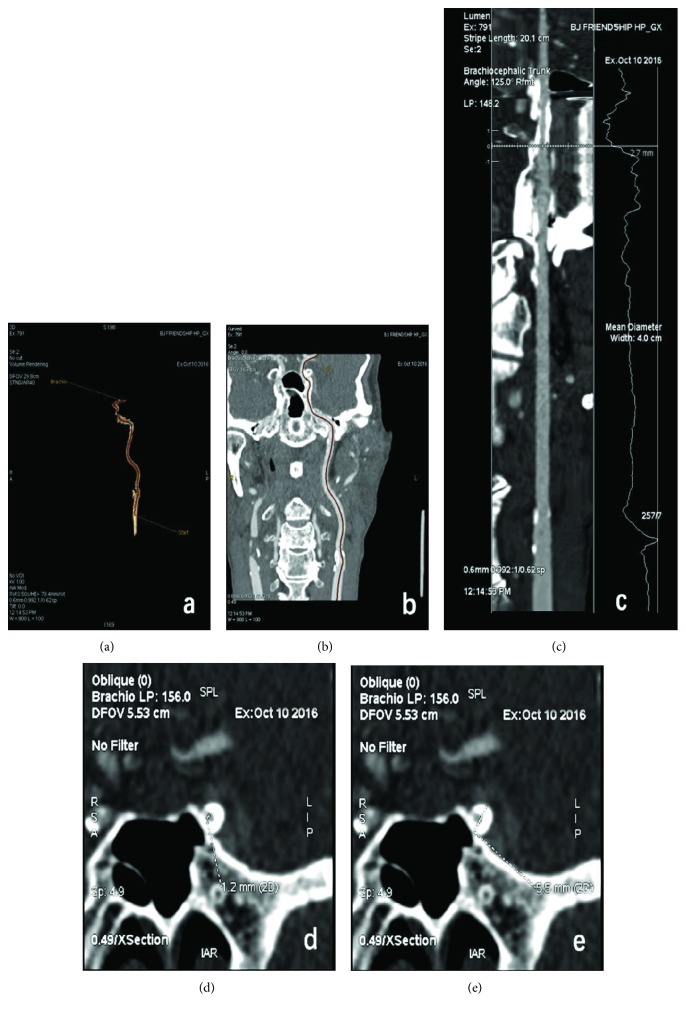
Head and neck CTA inspection. (a, b) The ICA trajectory. (c) Five segments of ICA blood vessels were measured in this study, and the measurement data of the narrowest blood vessel was selected. (d) shows the narrow vessel diameter, and (e) shows the normal vessel diameter; vascular stenosis degree % = normal vessel diameter − narrow vessel diameter, divided by the normal vessel diameter × 100%.

In accordance with the results of the ICA CTA examination, the patients were divided into four groups: the normal group (narrow rate 0), the mild stenosis group (ipsilateral carotid artery stenosis rate ≤ 29%), the moderate stenosis group (ipsilateral carotid artery stenosis rate of 30–69%), and the severe stenosis group (ipsilateral carotid artery stenosis rate of 70–100%) (Figure 1).

#### 2.2.2. Ultrasound Doppler Examinations

**Figure 2:**
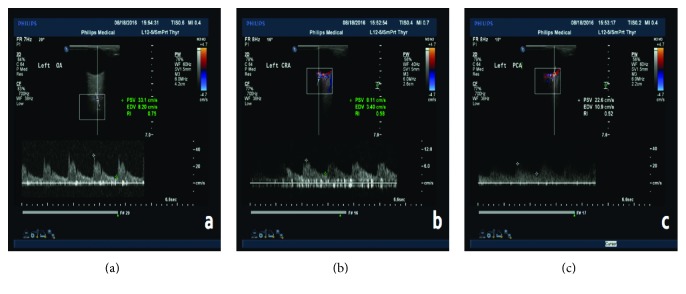
Ultrasound Doppler examinations of major ocular vessels and the measurement process of major vascular parameters. The parameters: peak systolic velocity (PSV), end diastolic velocity (EDV), and resistance indices (RI). (a) The measurement processes of the ophthalmic artery (OA): peak systolic velocity (PSV), end diastolic velocity (EDV), and resistance indices (RI). (b) The measurement processes of the central retinal artery: peak systolic velocity (PSV), end diastolic velocity (EDV), and resistance indices (RI). (c) The measurement processes of the posterior ciliary artery: peak systolic velocity (PSV), end diastolic velocity (EDV), and resistance indices (RI).

Blood flow velocities of the major ocular vessels were performed with a Philips iU 22 (PHILIPS, Netherland), with the use of linear 12–15 MHz transducers. All measurements were performed in the supine position and were taken by the same operator. The main parameters of ultrasound Doppler imaging are peak systolic velocity (PSV), end diastolic velocity (EDV), resistance indices (RI), and the calculation of the pulsation indices (PI) through the use of a formula. Three major ocular vessels (i.e., ophthalmic artery, central retinal artery, and posterior ciliary artery) were evaluated (Figure 2). In each vessel, peak systolic velocity (PSV), defined as the highest velocity of blood flow during the systolic phase of the cardiac cycle, and the end diastolic velocity (EDV), defined as the velocity of blood flow at the end of the diastolic phase of the cardiac cycle, are used [16]. PSV reflects the extent of vascular filling and blood supply, EDV reflects the blood perfusion situation of the distal tissue, and a numerical drop shows a shortage of distal blood supply. A decreased RI shows low resistance in the distal vascular bed and greater blood flow, while a decreased PI shows that the diastolic blood flow has increased.

#### 2.2.3. Ophthalmic Examinations

All patients received a fundus photograph and OCT examination.

**Figure 3:**
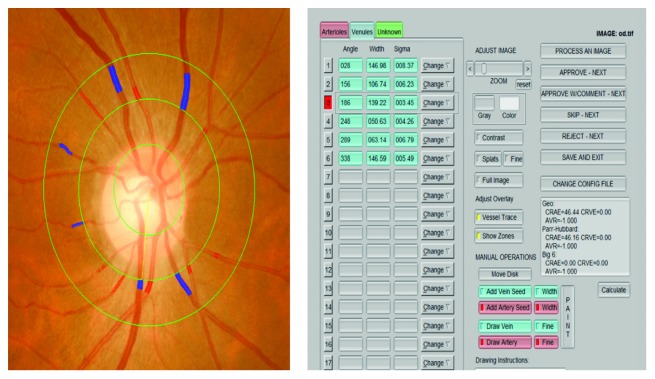
Retinal arteriolar and venular diameters. Semiautomated software (IVAN software, Australia) was used for the quantitative analysis. The software employs automatic optic disc detection and measures the calibre of six peripheral retinal arteriolar and venular vessel diameters up to 1 DD from the optic disc margin. The major parameters: central retinal artery equivalent (CRAE), central retinal vein equivalent (CRVE), and arteriolar-to-venular diameter ratio (AVR). Measurements were taken within an area (0.5–1.0 DD) centered on the optic disc.

*(1) Fundus Photographs.* Digital fundus photographs were obtained using nonmydriatic fundus photography. Binocular digital photographs were taken using a 45° high-resolution fundus camera (Kowa, Tokyo, Japan), and retinal fundus images were centered on the optic disc. The fundus photograph side with the better quality image was analysed, and double-blinded analyses were performed by two professionally trained ophthalmologists. A computer-based standardized protocol (IVAN software, Australia) automatically measures the diameter of arterioles and venules. This software employs automatic optic disc detection and measures the calibre of six peripheral retinal arteriolar and venular vessels with diameters up to 1-disc diameter (DD) from the optic disc margin; measurements were taken within an area (0.5–1.0 DD) centered on the optic disc. The central retinal artery equivalent (CRAE), central retinal vein equivalent (CRVE) and arteriolar-to-venular diameter ratio (AVR) are calculated by the revised Knudtson-Parr-Hubbard formula from an average of the biggest six vessels reflecting the estimated diameter of the central retinal artery and vein Ref (Figure 3).

**Figure 4:**
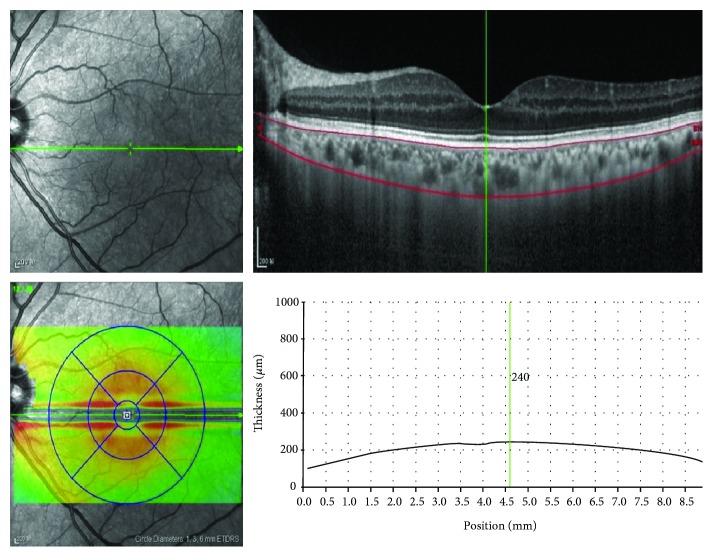
Subfoveal choroidal thickness (SFChT) measurements. For SFChT measurements, we manually moved the automatically segmented internal limiting membrane line to the choroidoscleral junction. Once we changed the automatically segmented line and set a vertical line perpendicular to the center of the fovea, the SFChT was automatically calculated.

*(2) OCT Examination and Choroidal Thickness Measurement.* A Heidelberg Spectralis device (Heidelberg Engineering, Heidelberg, Germany) was used to obtain SD-OCT images to measure the thickness of RNFL, inner retinal layers, and outer retinal layers. Coherence tomography with EDI-OCT was used in choroidal thickness measurements. The measurements were performed manually using the calliper function of the OCT device. The subfoveal choroidal thickness (SFChT) was defined as a vertical line perpendicular to the center of the fovea from the outer portion of the hyperreflective line of Bruch's membrane to the choroidoscleral interface (Figure 4). All OCT examinations were performed by the same experienced technician, and the SFChT measurements in the current study were performed by a retina specialist who was blinded to the group designations.

## 3. Statistical Analysis

Epidata was adopted to establish the database, and all data were entered twice. The data were statistically analysed using the one-way analysis of variance or Kruskal-Wallis test; gender differences and medical history were analysed using Pearson's or Fisher's exact chi-square tests. All statistical analyses were undertaken using SPSS 22.0 (SPSS Inc., Chicago, IL). We also analysed the effects of confounding factors on the final analysis parameters to balance the various confounding factors between groups.

Normality tests were conducted for all data in every group. One-way ANOVAs were used if the data obeyed normal distribution characteristics, and the intergroup least significant difference (LSD) method was applied. The statistical significance was set at *p* < 0.05. All tests should be understood and interpreted as constituting an exploratory data analysis in such a way that no previous power calculation or adjustments for multiple testing were made.

## 4. Ethics Statement

This study was approved by the local ethics committee of Beijing Friendship Hospital and was conducted in conformance with the Declaration of Helsinki (the 2013 revision), the guideline of the International Conference on Harmonisation of Good Clinical Practice, and the applicable Chinese laws. In addition, all participants provided written informed consent.

## 5. Results

A total of 219 patients were examined with a group comprised of 147 males and 72 females. The mean age of all included patients was 61.85 ± 7.86 years. The baseline information and characteristics of the patients are shown in Table 1. The results indicate there were no significant differences among the four groups in terms of age, gender, body mass index, or the prevalence of hypertension and hyperlipidemia. Thus, we have ignored their influence on the final analyses.

## 6. Retinal Vascular Diameter and the Ratio of the Artery and Vein

Table 2 shows that the central retinal artery equivalent (CRAE), central retinal vein equivalent (CRVE), and arteriolar-to-venular diameter ratio (AVR) followed a normal distribution among the four groups. The CRAE for each group was as follows: the normal group = 163.17 ± 16.97 *μ*m; the mild stenosis group = 157.39 ± 15.72 *μ*m; the moderate stenosis group = 154.72 ± 20.22 *μ*m; and the severe stenosis group = 152.80 ± 25.76 *μ*m (*p* = 0.073). The CRVE for each group was as follows: the normal group = 244.89 ± 22.42 *μ*m; the mild stenosis group = 235.10 ± 25.44 *μ*m; the moderate stenosis group = 243.50 ± 24.00 *μ*m; and the severe stenosis group = 244.25 ± 29.72 *μ*m (*p* = 0.188). The AVR for each group was as follows: the normal group = 0.66 ± 0.07; the mild stenosis group = 0.66 ± 0.07; the moderate stenosis group = 0.65 ± 0.08; and the severe stenosis group = 0.66 ± 0.07 (*p* = 0.738). There were no significant differences among the groups (Table 2).

## 7. Vascular Haemodynamic Parameters of Major Ocular Vessels

Table 3 shows the vascular haemodynamic parameters among the four groups. PSV and EDV of the central retinal artery and posterior ciliary artery in ICA stenosis groups were significantly lower than those of the normal group (central retinal artery: *p* < 0.01; posterior ciliary artery: *p* < 0.01, resp.). But PI and RI of the two arteries were not significantly different among the four groups (central retinal artery: *p* = 0.332, *p* = 0.446; posterior ciliary artery: *p* = 0.557, *p* = 0.856, resp.). The major parameters of OA were not significantly different among the four groups (*p* = 0.983, *p* = 0.934, *p* = 0.870, and *p* = 0.814, resp.).

## 8. The Retinal Thickness and the Subfoveal Choroidal Thickness

Results showed that the RNFL thickness, inner retinal layer thickness, the outer retinal layer thickness, and the SFChT followed a normal distribution among the four groups.

The RNFL thickness was as follows: the normal group = 11.60 ± 1.84 *μ*m; the mild stenosis group = 11.55 ± 1.78 *μ*m; the moderate stenosis group = 11.44 ± 1.42 *μ*m; and the severe stenosis group = 11.78 ± 1.52 *μ*m (*p* = 0.715). The inner retinal layer thickness was as follows: the normal group = 138.40 ± 23.54 *μ*m; the mild stenosis group = 135.12 ± 16.23 *μ*m; the moderate stenosis group = 132.77 ± 11.65 *μ*m; and the severe stenosis group = 139.35 ± 14.10 *μ*m (*p* = 0.100). There were no significant differences among the groups.

The outer retinal layer thickness was as follows: the normal group = 81.84 ± 8.54 *μ*m; the mild stenosis group = 79.00 ± 6.83 *μ*m; the moderate stenosis group = 78.90 ± 5.66 *μ*m; and the severe stenosis group = 77.37 ± 8.36 *μ*m (*p* = 0.030) (Table 4). The mean outer retinal layer thickness values of the normal eyes were significantly bigger than those of the other three ICA stenosis groups (*p* = 0.030).

The mean SFChT values of the ICA stenosis groups were significantly lower than those of normal eyes (normal group SFChT = 287.44 ± 53.52 *μ*m; mild stenosis group SFChT = 227.00 ± 50.32 *μ*m; moderate stenosis group SFChT = 217.74 ± 54.73 *μ*m; and severe stenosis group SFChT = 166.94 ± 51.95 *μ*m; *p* < 0.001) (Table 4).

## 9. Discussion

ICA stenosis is a relatively common clinical disease that is mostly caused by atherosclerosis. It is also an important cause of ischemic cerebral apoplexy. ICA stenosis can lead not only to nervous system diseases but also ischemic ocular lesions. Ocular haemodynamic changes have become an ophthalmological research focus in recent years.

Color Doppler imaging (CDI) can effectively evaluate the blood flow velocity of ophthalmic small blood vessels, such as the ophthalmic artery, the central retinal artery, and the posterior ciliary artery [17]. If the ICA is stenotic, the blood flow condition of the OA and its branches may be change. Kiseleva found that the blood flow of both the OA and central retinal artery with acute and chronic ischemic damage had been significantly reduced or disappeared. The maximum blood flow velocity had decreased by twofold, end diastolic blood flow had a fivefold decrease, and the resistance index increased 1.5 times in the systolic period. Significant carotid stenosis or occlusion may be accompanied by arteriosclerosis of the orbital arteries, leading to higher resistance in small orbital vessels, as suggested by Ho et al. [17]. When the degree of ICA stenosis is comparatively low, ocular ischemic changes are less prone to appear. However, with the severity of ICA stenosis increased, ocular ischemia may occur. Therefore, when patients have ocular ischemic changes, carotid artery stenosis may have developed to a certain extent.

The OA, which is the first branch of the ICA, branches to form the central retinal artery and the posterior ciliary arteries when it enters the orbital cavity through the optic canal. Because the central retinal artery and the long posterior ciliary arteries are branches of the OA, the central retinal artery is the only one that supplies blood to the inner layer of the retina while the posterior ciliary arteries supply blood to the outer layer of the retina and choroid. Therefore, any inadequate arterial perfusion disease can affect the choroidal and retinal microcirculation status and lead to decreased choroidal and retinal perfusion, which may then eventually cause ischemic ocular lesions.

Various causes of common carotid artery and ICA stenosis can lead to OA blood supply deficiencies, which then may cause OIS. The ocular ischemic diseases caused by ICA stenosis have been widely recognized in clinical studies. There are mainly four haemodynamic parameters we can analyse in the blood vessels, the PSV, EDV, RI and, through a formula, PI. The PSV can reflect blood vessel filling and blood supply strength, and is a reflection index of vasoconstriction and resistance conditions. Studies have shown that when the ICA is stenotic, ocular blood flow is significantly reduced, making the PSV decrease and peripheral resistance increase, which can lead to ischemic ocular disease.

Feng et al. [18] analysed and investigated the correlation of amaurosis fugax and carotid stenosis as well as posterior ocular blood vessel haemodynamic changes. This group showed that the blood flow velocity of the central retinal artery in amaurosis fugax patients is significantly reduced, while the resistance was normal. These results are in accordance with our findings. We found that the PSV and EDV of the central retinal artery and posterior ciliary artery were significantly lower in the patients with carotid artery stenosis than those in the control group. Moreover, reductions in PSV and EDV are consistent with the increased ICA stenosis. Presumably, ocular blood flow is controlled by local autoregulation, and in cases of ICA stenosis, it may be maintained by collateral circulation. Blood flow in the central retinal artery and posterior ciliary artery decreases significantly when severe ICA stenosis occurs. These blood flows may not be maintained by collateral circulation, and this can lead to a decreased EDV. However, there were no significant differences in haemodynamic indices in the OA between the ICA stenosis groups and the normal group in our study. The reason for this may be that, since the OA is the first branch of the ICA, blood vessels in the head and neck have strong collateral compensative capacity. With collateral vessel patency, ocular blood flow may be maintained by the expansion of blood vessels and self-correcting mechanisms, which may counteract reductions in blood perfusion and velocity caused by ICA stenosis, resulting in few overall changes.

According to our findings, a lack of blood flow in the central retinal artery can lead to retinal ischemic lesions. Furthermore, with an increased degree of ICA stenosis, there are significant reductions in the haemodynamic indices (PSV and EDV) of the central retinal artery. Our results showed that ICA stenosis can cause abnormal blood flow in the central retinal artery, but it is not clear how the related fundus microstructure changes. The posterior ciliary arteries supply blood to the outer layer of the retina and choroid, so any inadequate arterial perfusion can affect the choroid microcirculation status and lead to decreased choroid perfusion. Eventually, this may cause choroidal microstructural changes. Numerous prospective, randomized, and multicenter studies have been designed to evaluate choroidal thickness in normal and diseased states. However, no clear evidence has been found regarding the effect of ICA stenosis on outer retinal layer thickness and choroidal thickness. ICA stenosis is the most important cause of OIS. We analysed the inner retinal layer thickness, the outer retinal layer thickness and the choroidal thickness in patients with different severities of ICA stenosis. We found that the outer retinal layer thickness of the ICA stenosis eyes was significantly lower than that in the normal control group, but there were no significant differences of the inner retinal layer thickness among the groups. From this, we also determined that with an increased degree of ICA stenosis, the SFChT in the eye on the stenotic side was obviously decreased. This trend between SFChT changes and ICA stenosis suggests that carotid arterial stenosis can produce an abnormal retinal choroid blood supply.

Salazar et al. [19] investigated alterations in the choroid in hypercholesteraemic rabbits and concluded that the choroid was thicker in the presence of hypercholesterolaemia. Similarly, Wong et al. [20] reported that the mean SFChT was thicker among patients with hypercholesterolaemia. A previous study has reported on three cases of OIS patients who showed lower choroidal thickness in the affected eye [14]. Kim et al. [21] analysed 19 unilateral OIS patients and concluded that the SFChT and choroidal volume of OIS eyes were significantly less than that of normal eyes. These above results are in accordance with our results. Decreased choroidal circulation caused by carotid artery stenosis might affect the discordance of choroidal thickness.

We analysed all data in our study, the results showed that the outer retinal layer thickness and the SFChT of the ICA stenosis eyes were significantly lower than those in the normal group, and the decreased SFChT was consistent with the decreased blood flow of the posterior ciliary arteries. PSV and EDV decreases in the posterior ciliary arteries were closely related to the decrease in SFChT. This suggests that ICA stenosis can influence the blood flow of the posterior ciliary arteries, which results in an insufficient blood perfusion of choriocapillaris, alterations in the blood supply of the outer layers of retinal, and changes to the normal retinal metabolism, resulting in an oxygen-starved retina. Once these occur, choroid degenerative disease may appear in a vicious cycle over a long period of time. Thus, there is a close relationship between choroid degenerative disease and insufficient blood perfusion of the choriocapillaris, which might be an important risk factor in the development of choroid degenerative disease.

When the ICA is obstructed, blood flow to the brain is maintained by the opening of collateral channels between the terminal external carotid artery branches and the terminal branches of the OA. Dilation of the ECA-ICA collateral channels via the OA can cause many signs and symptoms including a delay/asymmetry of ECA pulses, angular, brow, and cheek pulses, and the frontal artery sign. Retrograde flow to the ICA occurs through these channels via the OA, resulting in hypoperfusion of the retinal and choroidal circulation [22]. This phenomenon may lead to compromised choroidal flow and result in photoreceptor dysfunction [23]. Structurally and functionally, normal choroidal vasculature is crucial for proper retinal function [23]. Our results also show that ICA stenosis can not only cause an abnormal haemodynamic status of the central retinal artery but can also affect the blood supply of the posterior ciliary arteries and lead to choroid atrophic lesions. When the ICA stenosis occurs, hypoperfusion of the outer retinal and choroidal circulation may happen early than the hypoperfusion of the inner retinal layers. However, it is unclear if there were any connections between choroidal ischemia and retinal ischemia and how they interact with each other. We lacked a large sample size study to confirm the relationship between the central retinal artery blood flow state and changes in the fundus microstructure. In addition, in the progress of OIS, no particular order has been found in the thickness changes of the choroidal and retinal changes.

The current research concerning SFChT was very limited, and the strength of our study is that it was the first to analyse the correlation between ocular haemodynamic parameters and SFChT. However, the sample size of this study was relatively small, which may have limited the statistical strength of the analysis and reduced our ability to perform correlational analyses for other OA branches and the fundus microstructure. Future studies should be performed with larger cohorts and longer follow-up periods to determine correlational relationships between retinal ischemia and choroidal ischemia. It will be also necessary to observe how the retina and choroid change with the progression of ICA stenosis by a serial evaluation. In conclusion, decreased choroidal circulation caused by carotid artery stenosis may significantly affect the discordance of choroidal thickness.

## 10. Conclusion

The present study revealed that the mean PSV and EDV in the central retinal artery and posterior ciliary artery and the mean SFChT and outer retinal layers thickness of ICA eyes were significantly lower than those of normal eyes. ICA stenosis can not only cause an abnormal haemodynamic status of the central retinal artery but can also affect the choroidal haemodynamics and decreased choroidal circulation then leading to the discordance of the SFChT and the outer retinal layer thickness.

## Figures and Tables

**Table 1:** Characteristics of participants.

Characteristic	Normal group	Mild stenosis group	Moderate stenosis group	Severe stenosis group	*p* value
Gender (M/F)	29/43 (67.4%)	32/49 (65.3%)	52/78 (66.7%)	34/49 (69.4%)	*p* = 0.980^∗^
Ages (years)	59.21 ± 5.34	62.20 ± 9.10	62.97 ± 7.07	62.04 ± 9.16	*p* = 0.085^†^
BMI (kg/m^2^)	24.48 ± 2.63	25.22 ± 3.44	25.42 ± 3.06	25.41 ± 3.69	*p* = 0.441^†^
Medical history					
Hypertension/*n* (%)	32/43 (74.4%)	37/49 (75.5%)	58/78 (74.4%)	38/49 (77.6%)	*p* = 0.985^∗^
Hyperlipidemia/*n* (%)	21/43 (48.8%)	24/49 (50.0%)	39/78 (50.0%)	24/49 (48.9%)	*p* = 1.000^∗^

^∗^Pearson's or Fisher's exact test of chi-square tests.

^†^ *p* values were calculated using the one-way analysis of variance.

**Table 2:** Retinal vascular diameter and arteriolar-to-venular diameter ratio (AVR).

	Normal group	Mild stenosis group	Moderate stenosis group	Severe stenosis group	*p* value
CRAE (*μ*m)	163.17 ± 16.97	157.39 ± 15.72	154.72 ± 20.22	152.80 ± 25.76	*p* = 0.073^†^
CRVE (*μ*m)	244.89 ± 22.42	235.10 ± 25.44	243.50 ± 24.00	244.25 ± 29.72	*p* = 0.188^†^
AVR	0.66 ± 0.07	0.66 ± 0.07	0.65 ± 0.08	0.66 ± 0.07	*p* = 0.738^†^

CRAE: central retinal artery equivalent; CRVE: central retinal vein equivalent; AVR: arteriolar-to-venular diameter ratio.

^†^ *p* values were calculated using the one-way analysis of variance.

**Table 3:** Vascular haemodynamic parameters of major ocular vessels.

Vascular	Indicators	Normal group	Mild stenosis group	Moderate stenosis group	Severe stenosis group	*p* value
OA	PSV	34.40 ± 14.91	34.38 ± 13.15	35.20 ± 13.54	34.32 ± 16.86	*p* = 0.983^†^
	EDV	7.55 (5.53, 10.40)	8.01 (5.96, 10.44)	7.66 (5.40, 10.20)	7.30 (5.70, 10.00)	*p* = 0.934^‡^
	RI	0.76 (0.73, 0.80)	0.77 (0.72, 0.80)	0.76 (0.72, 0.80)	0.74 (0.64, 0.84)	*p* = 0.870^‡^
	PI	1.21 (1.07, 1.34)	1.26 (1.13, 1.33)	1.24 (1.13, 1.35)	1.18 (0.95, 1.43)	*p* = 0.814^‡^

CRA	PSV	11.80 (9.20, 15.80)	11.60 (8.86, 15.80)	10.10 (8.28, 13.86)	8.90 (6.92, 11.40)	*p* < 0.001^‡^
	EDV	3.80 (2.80, 5.30)	4.00 (2.63, 5.25)	3.50 (2.40, 4.90)	2.61 (1.87, 3.60)	*p* = 0.001^‡^
	RI	1.20 ± 0.16	1.00 ± 0.16	1.20 ± 0.16	1.20 ± 0.16	*p* = 0.332^†^
	PI	1.05 (0.95, 1.14)	1.00 (0.91, 1.11)	1.02 (0.88, 1.14)	1.04 (0.93, 1.19)	*p* = 0.446^‡^

PCA	PSV	16.43 ± 5.28	15.01 ± 4.64	14.11 ± 4.37	12.38 ± 4.32	*p* < 0.001^†^
	EDV	5.30 (4.06, 6.50)	4.93 (4.00, 6.30)	4.50 (3.50, 5.54)	3.70 (2.74, 4.64)	*p* < 0.001^‡^
	RI	0.66 ± 0.07	0.66 ± 0.08	0.67 ± 0.07	0.68 ± 0.08	*p* = 0.557^†^
	PI	1.00 ± 0.17	1.00 ± 0.18	1.01 ± 0.15	1.02 ± 0.25	*p* = 0.856^†^

OA: ophthalmic artery; CRA: central retinal artery; PCA: posterior ciliary artery; PSV: peak systolic velocity; EDV: end diastolic velocity; RI: resistance indices; PI: pulsation indices.

^†^ *p* values were calculated using the one-way analysis of variance.

^‡^ *p* values were calculated using the Kruskal-Wallis test.

**Table 4:** Retinal thickness and the subfoveal choroidal thickness.

	Normal group	Mild stenosis group	Moderate stenosis group	Severe stenosis group	*p* value
RNFL (*μ*m)	11.60 ± 1.84	11.55 ± 1.78	11.44 ± 1.42	11.78 ± 1.52	*p* = 0.715^‡^
Inner retinal layer (*μ*m)	138.40 ± 23.54	135.12 ± 16.23	132.77 ± 11.65	139.35 ± 14.10	*p* = 0.100^†^
Outer retinal layer (*μ*m)	81.84 ± 8.54	79.00 ± 6.83	78.90 ± 5.66	77.37 ± 8.36	*p* = 0.030^†^
SFChT (*μ*m)	283.95 ± 54.64	227.00 ± 50.32	217.74 ± 54.73	166.94 ± 51.95	*p* < 0.001^†^

RNFL: retinal nerve fiber layer; SFChT: subfoveal choroidal thickness.

^†^ *p* values were calculated using the one-way analysis of variance.

^‡^ *p* values were calculated using the Kruskal-Wallis test.
